# ASO Author Reflections: Assessing the Impact of Neoadjuvant Therapy: A Real View Perspective

**DOI:** 10.1245/s10434-020-08403-y

**Published:** 2020-03-29

**Authors:** Sivesh K. Kamarajah, Alexander W. Phillips

**Affiliations:** 1 Northern Oesophagogastric Unit, Royal Victoria Infirmary, Newcastle University Trust Hospitals, Newcastle upon Tyne, UK; 2 School of Medical Education, Newcastle University, Newcastle upon Tyne, UK

## Past

Neoadjuvant therapy improves long-term survival by providing locoregional disease control and reducing the risk of long-term recurrence. The most important prognostic factor of survival after neoadjuvant therapy followed by surgery is the burden of lymph node involvement. Several trials have demonstrated improved long-term survival with neoadjuvant therapy; however, there are little data evaluating adenocarcinoma and squamous cell carcinoma (SCC) and difference in outcomes for similar pathological stage with and without neoadjuvant treatment. This study^1^ demonstrated pathological stage provides a better estimate of prognosis compared with clinical stage. Downstaged patients may have an improved outcome over those with comparable pathological stage who did not receive neoadjuvant treatment.

## Present

These results corroborate what has been found in other recent studies^2^,^3^ that disease downstaging with neoadjuvant treatment is associated with better overall survival. This has led to the acceptance of neoadjuvant therapy for locally advanced esophageal cancer as the standard of care. However, it is apparent that not all patients respond to neoadjuvant treatment to the same degree and the observed impact could be used to tailor adjuvant treatment. In addition, there appeared to be better survival in patients who received neoadjuvant therapy compared with similarly staged neoadjuvant-naïve patients. This was apparent through all stages for SCC, and evident in more advanced adenocarcinoma.

## Future

This study reinforces the understanding that post-neoadjuvant stage influences prognosis. While some may advocate ‘complete’ restaging prior to progressing to surgery, induction therapy is known to potentially impact on the reliability of staging modalities. It may also be important to consider the post-neoadjuvant stage when deciding on the merit of adjuvant treatment. There has been some suggestion that adjuvant treatment may confer some benefit in node-positive patients with adenocarcinoma who received neoadjuvant chemoradiotherapy, and it already forms part of the standard of treatment in patients receiving MAGIC protocol chemotherapy.^4^ However, the ability to predetermine the impact of neoadjuvant therapy on disease stage based on specific biological factors of a tumor may further individualize oncological therapy, as demonstrated in colon cancer.^5^ This will guide informed decision among patients and clinicians moving forwards.
